# Covariant perfusion patterns provide clues to the origin of cognitive fluctuations and attentional dysfunction in Dementia with Lewy bodies

**DOI:** 10.1017/S1041610213001488

**Published:** 2013-12

**Authors:** John-Paul Taylor, Sean J. Colloby, Ian G. McKeith, John T. O'Brien

**Affiliations:** 1 Institute for Ageing and Health, Campus for Aging and Vitality, Newcastle University, Newcastle upon Tyne, UK; 2 Department of Psychiatry, University of Cambridge, Addenbrooke's Hospital, UK

**Keywords:** attention, Alzheimer’s disease, single photon emission computed tomography, SPECT, imaging

## Abstract

**Background:**

Fluctuating cognition (FC), particularly in attention, is a core and defining symptom in dementia with Lewy bodies (DLB) but is seen much less frequently in Alzheimer's dementia (AD). However, its neurobiological origin is poorly understood. The aim of our study was therefore to characterize perfusion patterns in DLB patients that are associated with the severity and frequency of FC as measured both clinically and using objective neuropsychological assessments.

**Methods:**

Spatial covariance analyses were applied to data derived from single photon emission computed tomography (SPECT) HMPAO brain imaging in 19 DLB and 23 AD patients. Patients underwent clinical assessment of their FC and cognitive function as well as objective testing of their attention.

**Results:**

Covariant perfusion principal components (PCs) were not associated with either FC or cognitive or attentional measures in AD. However, in DLB patients, the second PC (defined as DLB-cognitive motor pattern, DLB-PCI2) which was characterized by bilateral relative increases in cerebellum, basal ganglia, and supplementary motor areas and widespread bilateral decreases in parietal regions, positively correlated with poorer cognitive function, increased FC and worse attentional function measured both clinically and neurophysiologically (p < 0.05) as well as with the severity of bradykinesia (p = 0.04).

**Conclusions:**

FC in DLB appears distinct from those seen in AD, and likely to be driven by internal neurobiological perturbations in brain circuitry as evidenced using spatial covariance analyses of cerebral perfusion. FC and certain aspects of attentional dysfunction in DLB may, in part, depend upon both distributed motor and non-motor networks.

## Introduction

Dementia with Lewy bodies (DLB) is the second most common form of degenerative dementia in older adults accounting for 15%–20% of neuropathologically defined cases.

A core symptom of DLB is that of fluctuating cognition (FC), which can affect up to 90% of patients (McKeith *et al*., 2005). Clinically, these spontaneous alterations in arousal, attention, and cognition can have significant impacts on patient function (Ballard *et al*., 2001). Qualitatively, FC in DLB appears to be distinct from the less frequently seen fluctuations in other dementias such as Alzheimer's disease (AD), with an interruption of awareness which is often associated with transient episodes of confusion and communicative difficulties (McKeith *et al*., 2005). Remission to near-normal cognitive function can occur spontaneously in the absence of clear environmental triggers, suggesting that FC in DLB is internally driven in contrast to AD, where FC may be more dependent upon changes in the environment (Bradshaw *et al*., 2004).

Clinical assessment of FC can be performed using a number of semi-standardized clinical scales such as the clinical assessment of fluctuations (CAF) scale (Walker *et al*., 2000a) or the Mayo fluctuations Questionnaire (Ferman *et al*., 2004). Objective measures of FC have included performance variability in attentional tasks including the choice reaction time (CRT) (Walker *et al*., 2000a). However, despite the prominence and pervasiveness of FC in DLB, there is no clear biomarker of FC and the underlying etiology of FC in DLB is poorly understood.

DLB patients, on electroencephalography (EEG), tend to display increased slow wave activity posteriorly compared to AD patients (Bonanni *et al*., 2008) and in DLB patients the EEG frequency fluctuates in periodic patterns, particularly posteriorly, in the delta–theta/prealpha or theta/prealpha/alpha range with these abnormalities positively correlating with the frequency and severity of clinically observed FC (Walker *et al*., 2000b). However, which brain areas contribute to FC is unknown and no obvious structural brain changes have been associated with FC in DLB. Whitwell *et al*. (2007) observed volume reductions in the hypothalamus of DLB and they suggested this might relate to altered arousal and attention in DLB although this study did not make any direct link between this structural change and the presence or severity of FC. Rather it is likely that FC arises from functional and network perturbations rather than overt structural abnormalities. In support of this, a recent functional blood-oxygen-level-dependent (BOLD) resting state network analysis found that reductions in functional connectivity between right frontal and parietal in DLB patients were associated with FC (Franciotti *et al*., 2013). In addition, clinicopathological data have demonstrated dopaminergic and cholinergic changes in thalamic areas in DLB patients with disturbed consciousness/arousal compared to those without (Pimlott *et al*., 2006; Piggott *et al*., 2007) and consistent with this finding is the observation of increased FC and increased thalamic perfusion on HMPAO single photon emission computed tomography (SPECT) in Lewy body dementia patients (O'Brien *et al*., 2005). In particular, the cholinergic system which is widely distributed in brain and differentially more affected in DLB than AD may have a role; pharmacological evidence has demonstrated that anticholinergic drugs can induce a symptom profile of altered arousal (Perry *et al*., 1999) that is similar to FC seen in DLB and, by contrast, cholinesterase inhibitors (CHEI) can significantly improve FC and attentional function in DLB (McKeith *et al*., 2000; Wesnes *et al*., 2002).

Taking these data together with known EEG changes in DLB, we hypothesized that FC is likely to be associated with spatially distributed networks, perhaps modulated by cholinergic function, rather than focally located in any single brain structure. One way to test this hypothesis is the application of multivariate network approaches and one specific technique is that of voxel spatial covariance analysis (SCA) called scaled subprofile model (Gene and Alexander, 1994), which is an extension of principal component analysis (PCA), which generate a series of PCA eigenimages of brain uptake representing significant sources of variance in the data that may also reflect specific disease characteristics.

Use of these analyses applied to positron emission tomography (PET) and SPECT images is not without precedent in Lewy body disease (Eckert *et al*., 2007b): in Parkinson's disease (PD), SCAs have helped characterize the relationship between disease-specific covariant metabolic networks and cognitive and motor dysfunction as well as providing a biometric probe for therapeutic response, for example, to a dopaminergic medication (Eckert *et al*., 2007a; Hirano *et al*., 2009). In addition, more recently we have shown that a multivariate approach can usefully discriminate between DLB and AD with high sensitivity and specificity (Colloby *et al*., 2013).

In the present study, we applied SCA to HMPAO perfusion SPECT data in a cohort of DLB and AD patients who underwent comprehensive clinical and cognitive assessments and our primary aim was to identify any spatial covariant perfusion principal component images (PCIs) related to FC, attentional dysfunction and, in DLB, to the severity of Parkinsonism given the prior evidences of covariant networks associated with motor dysfunction in PD (Eckert *et al*., 2007a). If such PCIs were present and associated with FC, our secondary aim was to clarify if these patterns were differentially expressed in those taking CHEI compared to those not on these agents.

## Methods

### Patients

Dementia patients (23 AD and 19 DLB) were recruited from a local community-dwelling population of patients who had been referred to geographically based old age psychiatry and neurology services. The study was approved by the local research ethics committee and UK Department of Health's Administration of Radioactive Substances Advisory Committee (ARSAC). All patients, as well as the nearest relative for patients, gave informed written consent.

Patients underwent detailed physical, neurological, and neuropsychiatric examinations, which included history, mental state, and physical examination. Investigations included routine hematology and biochemistry screening, thyroid function tests, syphilis serology, vitamin B12 and folate levels, chest X-ray, and head CT scan.

Diagnosis of dementia type was made independently by consensus between three experienced senior clinicians using NINCDS-ADRDA criteria for AD (McKhann *et al*., 1984) and the international workshop criteria for DLB (McKeith *et al*., 1996). Sixteen of the 19 DLB patients also had clinical SPECT Fluoropropyl-Carbomethoxy-Iodophenyl-Tropane (FP-CIT) dopamine transporter scans to assist with diagnosis (abnormal in 12/16). In addition, neuropathological confirmation of diagnosis according to international criteria was available in four out of 23 AD patients (four definite AD) and in seven out of 19 DLB patients (six definite DLB and one mixed DLB/AD pathology). FP-CIT imaging was abnormal in 6/7 of the neuropathologically confirmed DLB cases.

### Assessments

Patients underwent a range of clinical assessments. Primary clinical variables in patients which were used to identify salient covariant perfusion networks associated with cognitive and attentional function included: Cambridge Cognitive Examination (CAMCOG), a marker of global cognitive function; Unified Parkinson's Disease Rating Scale Part III (UPDRS), a global marker of the severity of extrapyramidal signs; the CAF, as a clinical measure of FC (Walker *et al*., 2000a); CRT, a measure of information processing speed in a visual stimulus discrimination task and the variability (standard deviation) of this task (CRT(SD)) as an objective measure of FC (Walker *et al*., 2000a). Secondary clinical variables which were examined in terms of correlation with covariant perfusion networks included the Mini-Mental State Examination (MMSE), the CAMCOG subscales scores of orientation, attention, and calculation (domains dependent on attentional function); and the bradykinesia subscale score from the UPDRS given that bradykinetic-rigid motor profile (rather than tremor dominant) is more typically seen in DLB (Aarsland *et al*., 2001).

### 99mTc-exametazime (HPMAO) SPECT imaging

Patients were scanned using a triple-headed rotating gamma camera (Picker 3000XP) fitted with a high-resolution fan-beam collimator, approximately 10 minutes after a bolus intravenous injection of 500 MBq of 99mTc-exametazime (Ceretec, GE Healthcare, also referred to as HMPAO, GE Healthcare, Buckinghamshire, UK). One hundred and twenty 15-second views over a 360-degree orbit were acquired on a 128 × 128 matrix with a pixel and slice thickness of 3.5 mm. Imaging time was 30 minutes. Images were reconstructed using ramp-filtered backprojection with a Butterworth filter (order 13, cut-off 0.2 cycles cm^−1^), then resampled to a 64 × 64 matrix containing 4.0 mm cubic voxels. Axial resolution was 12 mm full width at half maximum (FWHM) and the reconstructed images were corrected for gamma-ray attenuation using Chang's algorithm (uniform attenuation coefficient, μ = 0.11 cm^−1^). Scatter correction was not applied.

### Spatial pre-processing

All images were spatially normalized using an affine transform (12 parameters) to match a 99mTc-exametazime SPECT template in standard MNI (Montreal Neurological Institute: http://www2.bic.mni.mcgill.ca/) space using FMRIB's linear image registration tool (FLIRT: http://www.fmrib.ox.ac.uk/fsl/flirt/index.html). Generation of the template image has been previously described (Colloby *et al*., 2008). Subsequent to this, images were visually inspected to ensure accuracy of registrations. A 16-mm FWHM 3D Gaussian filter was then applied to the registered scans, producing spatially smoothed images. Finally, scans were intensity scaled to their mean whole-brain uptake defined from a SPECT binary mask image.

### Multivariate spatial covariance analysis

Voxel-based SCA was applied separately to the pre-processed images of the AD and DLB dementia cohorts using the generalized covariance analysis software suite (http://www.nitrc.org/projects/gcva_pca/) (Habeck *et al*., 2005). A SPECT binary mask image defined the brain volume subspace for voxel-wise analyses and this generated a series of eigenimages/PCIs that were organized in a decreasing order of variance for each subsequent eigenimage. Voxels participating in each eigenimage may have either positive or negative weights, where the weights express the strength of interaction between voxels that contribute to the eigenimage. Voxels with positive and negative weighting can be interpreted as exhibiting concomitant increased and decreased activity/blood flow, respectively. Once calculated, eigenimage weights were fixed and equal for all patients within the respective dementia cohort. The degree to which a patient expressed each eigenimage was by means of a subject scaling factor SSF_i_, where a higher SSF score represents more concurrent increased flow of voxels with positive weights and more concurrent decreased flow of voxels with negative weights.

Disease-specific individual SSF expressions on each eigenimage, which contributed more than 10% of the variance, were correlated against the primary outcome measures; PCIs with significant correlations with at least one of these parameters were then analyzed further against our secondary measures.

Eigenimages were Z-thresholded at |Z| ≥ 1.64 (p ≤ 0.05), and the derived PCI topographic perfusion networks also underwent bootstrap resampling to determine significant local maxima which were contributory to the overall network. Localization of network regions in PCIs, which were significantly associated with clinical parameters, was further assessed using the MNI brain atlases within fslview (http://fsl.fmrib.ox.ac.uk/fsl/fslview/).

### Statistics

Patient expressions of the perfusion PCs were exported into the Statistical Package for Social Sciences software (SPSS ver. 19.0, http://www-01.ibm.com/software/analytics/spss/products/statistics/) for analysis. Normality of continuous variables was assessed using visual inspection of histograms and application of the Shapiro–Wilk test. Demographic and clinical data between groups were compared using student *t*-tests. In the case of reaction time variables, these data were positively skewed and thus were log-transformed to allow for parametric analyses. Categorical data were analyzed using χ^2^ tests.

Correlations between imaging data and clinical variables were determined using Pearson r. Consideration was then given to potential confounders including age, sex, CHEI use (score 0 = no use, score 1 = use of CHEI), and UPDRS scores (for reaction-time based tasks), if they correlated with imaging and clinical variables of interest (threshold p < 0.10). The confounder of disease severity was examined by considering duration of illness. In addition for measures of FC and attention (clinical and objective), the effect of global cognitive impairment (MMSE score) was also examined as a covariate. Potential confounder variables (p < 0.10) were then entered into a stepwise multiple regression models to assess their effect on the relationship between any PCI expression and dependent variables.

## Results

Patient demographic characteristics are reported in Table 1. AD and DLB patients were broadly matched in terms of age, gender, cognitive impairment, disease duration, and CHEI use. As expected, DLB patients had increased UPDRS and CAF scores and more attentional impairments compared to AD. CAF scores in DLB correlated with the objective measures of attention (CRT, r = 0.47, p = 0.04; CRT(SD), r = 0.54, p = 0.02).

**Table 1.** Demographic and clinical characteristics of patients

	ad	dlb	p-value
*N*	23	19	–
Gender (M:F)	11:12	12:7	0.37*
Age (yrs)	78.8 ± 6.0	76.2 ± 8.0	0.25
MMSE (max. 30)	17.5 ± 4.8	16.2 ± 5.6	0.77
CAMCOG (max. 107)	59.8 ± 15.8	63.8 ± 13.9	0.40
UPDRS III (max. 108)	5.8 ± 6.1	23.2 ± 13.8	<0.001
Disease duration (months)	33.2 ± 18.9	33.6 ± 26.0	0.96
CAF	0.3 ± 1.0	3.7 ± 3.5	<0.001
Choice reaction time (CRT)	6.41 ± 0.23	6.93 ± 0.49	<0.001
Choice reaction time standard deviation (CRT(SD))	5.10 ± 0.48	5.93 ± 0.74	<0.001
CHEI use (yes:no)	2:17	5:14	0.41*
L-dopa or equivalent use (yes:no)	0:23	2:17	N/A

Values expressed as (mean ± 1 standard deviation).

*χ^2^ value (df = 1).

AD = Alzheimer's disease; DLB = dementia with Lewy bodies; MMSE = Mini-Mental State Examination; CAMCOG = Cambridge Cognitive Examination; UPDRS III = Unified Parkinson's Disease Rating Scale (motor subsection); CAF = Clinical assessment of fluctuations; CHEI = cholinesterase inhibitor.

Note that reaction time data (milliseconds) reported have been log transformed.

### Disease-specific covariant perfusion patterns

**Figure 1.**
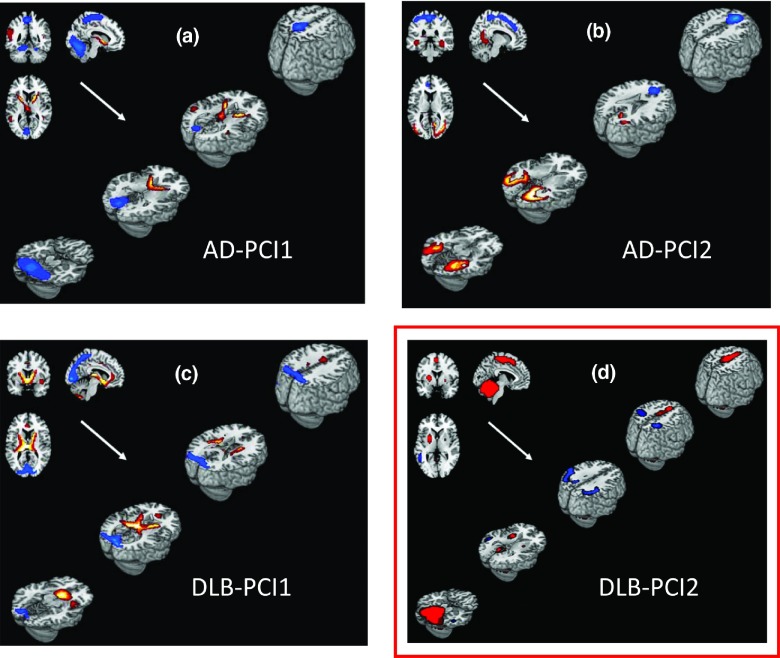
Covariant perfusion patterns in Alzheimer's disease (AD) and dementia with Lewy bodies (DLB) cohort contributing more than 10% of the variance superimposed onto structural MRI template images. Images show concomitant increase (red) and decrease (blue) regional cerebral blood flow (thresholded at |Z| ≥ 1.64, p ≤ 0.05). PCI = principal component image. Note only DLB-PCI2 correlated with clinical variables (boxed in red).

Covariant perfusion PCIs were derived separately for AD and DLB cohorts; the first two PCs accounted for variance greater than 10% (AD-PCI1, 18.7% and AD-PCI2, 13.9%; Figures 1a and b). In the DLB cohort, the first two PCs similarly accounted for variance greater than 10% (DLB-PCI1, 16.7% and DLB-PCI2, 15.3%; Figures 1c and d). AD-PCI1 and DLB-PCI1 demonstrated shared covariance perfusion patterns in selected brain regions in bilateral thalami and putamen (concomitant increases) as well as bilateral precuneus and left occipital lobe (concomitant decreases). Other regions specific to the AD-PCI1 pattern were bilateral pre- and post central gyrus, cerebellum (covariant decreases in perfusion), and left temporal lobe (covariant increases). Likewise, regions specific to the DLB-PCI1 pattern included anterior cingulate and bilateral caudate (covariant increases). AD-PCI2, in contrast, exhibited an approximate bilateral superior–inferior pattern with concomitant increased perfusion in inferior temporal and occipital lobe and concomitant decreased perfusion in anterior cingulate and pre and post central structures.

AD-PCI1, AD-PCI2, and DLB-PCI1 did not correlate significantly with our primary clinical variables (Table 2), and were therefore not analyzed in further detail. However, DLB-PCI2 was strongly associated with our primary cognitive, attentional, and FC measures (CAMCOG, CRT, CRT(SD), and CAF) and was therefore selected for further investigation. Table 3 presents brain regions having a significant contribution to the DLB-PCI2 covariance pattern. Concomitant increased perfusion was observed in motor networks, parietal and temporo-parietal cortices bilaterally, cerebellar hemispheres, particularly the midline and vermis, extending into the midbrain and bilateral supplementary motor areas (SMA) and putamen (although relatively smaller on right compared to left). Unilateral increases were also evident in left lingual gyrus. Concomitant decreases also occurred bilaterally in parietal cortex affecting inferior parietal, angular gyri, and supramarginal areas as well as in superior and middle temporal cortices. There was also a suggestion that left secondary visual areas were involved in the pattern.

**Table 2.** Pearson correlations between principal component image (PCI) expression and primary clinical variables

	ad		dlb	
	pci1	pci2	pci1	pci2
CAMCOG	0.11	0.08	0.08	−0.50*
CAF	0.01	−0.11	–0.34	0.59**
CRT	−0.29	0.11	−0.28	0.60**
(CRT(SD))	−0.30	0.16	−0.24	0.62**
UPDRS	−0.18	0.22	−0.13	−0.37

AD = Alzheimer's disease; DLB = dementia with Lewy bodies; CAMCOG = Cambridge Cognitive Examination; CAF = Clinical assessment of fluctuations; CRT = choice reaction time; CRT(SD) = choice reaction time variability (standard deviation); UPDRS III = Unified Parkinson's Disease Rating Scale (motor subsection).

*p < 0.05; **p < 0.01.

**Table 3.** Brain regions with significant contributions to the DLB cognitive motor pattern (DLB-PCI2). Regions reported had significant clusters (p < 0.05) with a minimum of ten voxels per cluster with localization of cortical and basal ganglia areas derived from Marsbar toolbox (MARSeille Boîte À Région d'Intérêt) and brainstem areas from wfupickatlas

region (z score ≥ 1.64)	cluster size	region (z score ≤ −1.64)	cluster size
Cerebellum_4_5_L	140	Angular_R	24
Cerebellum_4_5_R	36	Angular_L	45
Cerebellum_6_L	166	Occipital_Mid_L	14
Cerebellum_6_R	81	Parietal_Inf_L	85
Cerebellum_7_L	52	Parietal_Inf_R	21
Cerebellum_7_R	45	SupraMarginal_L	73
Cerebellum_Crus1_L	83	Temporal_Inf_R	14
Cerebellum_Crus1_R	111	Temporal_Mid_L	56
Fusiform_L	29	Temporal_Mid_R	27
Lingual_L	60	Temporal_Sup_L	36
Midbrain	42	Temporal_Sup_R	14
Parahippocampal_L	29		
Pons	24		
Putamen_L	42		
Supp_Motor_Area_L	112		
Vermis_10	14		
Vermis_3	29		
Vermis_4_5	63		
Vermis_6	31		
Vermis_7	24		
Vermis_8	30		
Vermis_9	17		

### Association between PCI2 expression in DLB patients and clinical variables

DLB-PCI2 correlated significantly with a number of the secondary clinical variables (Table 4). MMSE as well as CAMCOG subscale scores correlated negatively with the expression of DLB-PCI2, although the CAMCOG executive subscale was not significantly associated with DLB-PCI2.

**Table 4.** Univariate and multivariate associations between dementia with Lewy body (DLB) principal component image (PCI) 2 and clinical variables. Values expressed as (mean ± 1 standard deviation)

clinical variable	univariate r	p-value	clinical/disease covariates (p < 0.10)	multivariate analysis
Cognitive				
MMSE	−0.57	0.01	–	–
CAMCOG (total)*	−0.50	0.04	–	–
CAMCOG (executive)	−0.37	0.13	Age, r = –0.43, p = 0.08	F = 4.32, r = 0.60; age, beta = –0.34; p = 0.03; DLB-PCI2, beta = –2.45, p = 0.06
CAMCOG (memory)	−0.48	0.04	–	–
CAMCOG (orientation)	−0.58	0.009	–	
			–	–
CAMCOG (attention)	−0.54	0.02	–	–
CAMCOG (calculation)	−0.59	0.008	–	–
Cognitive fluctuations				
Clinical assessment of fluctuations*	0.59	0.009	CHEI, r = –0.46, p = 0.05	F = 8.86, r = 0.59; CHEI, beta = –0.15, p = 0.59; DLB-PCI2, beta = 2.07, p = 0.008
Objective attention tests				
Choice reaction time (CRT)*	0.60	0.007	Disease duration, r = 0.68, p = 0.005; CHEI, r = –0.47, p = 0.04; UPDRS, r = 0.59, p = 0.008; MMSE, r = –0.61, p = 0.008	F = 52.79, r = 0.98; disease duration, beta = 0.01, p < 0.001; CHEI, beta = 0.05, p = 0.62; UPDRS, beta = 0.01, p = 0.001; MMSE, beta = –0.03, p = 0.02; DLB-PCI2, beta = 0.16, p = 0.004
Choice reaction time standard deviation (CRT(SD))*	0.62	0.005	Disease duration, r = 0.48, p = 0.07; CHEI, r = –0.50, p = 0.03; UPDRS, r = 0.64, p = 0.003; MMSE, r = –0.67, p = 0.002	F = 24.90, r = 0.95; disease duration, beta = 0.01, p = 0.05; CHEI, beta = 0.09, p = 0.54; UPDRS, beta = 0.02, p = 0.009; MMSE, beta = –0.06, p = 0.006; DLB-PCI2, beta = 0.24 p = 0.02
Parkinsonism				
UPDRS III motor subscale score*	0.36	0.13	–	–
Bradykinesia subscale score	0.48	0.04	–	–

*Primary clinical variables of interest.

AD = Alzheimer's disease; DLB = dementia with Lewy bodies; MMSE = Mini-Mental State Examination; CAMCOG = Cambridge Cognitive Examination; UPDRS III = Unified Parkinson's Disease Rating Scale (motor subsection); CHEI = cholinesterase inhibitor (for regression purposes non-use score = 0, use of agent score = 1).

DLB-PCI2 was positively associated with UPDRS bradykinesia subscale score although not with the overall UPDRS score.

#### Effect of confounder variables

A number of confounder variables (age, disease duration, UPDRS, CHEI use, and MMSE scores) were significantly associated with clinical measures, although inclusion of these in a multivariate analysis did not significantly affect the relationship between DLB-PCI2 and the clinical variables in question (Table 4). Nevertheless, we separately considered the importance of CHEI use given its significant known effect on attention and FC. Our data confirmed (Supplementary Table 1, available as supplementary material attached to the electronic version of this paper at www.journals.cambridge.org/jid_IPG) that DLB patients on CHEI had less FC as measured on the CAF and faster CRT and lower CRT(SD) (p ≤ 0.006). In contrast, patients taking CHEI versus those not on these agents had similar global and subscale cognitive measures and Parkinsonism (p ≥ 0.26). Importantly however, DLBs who were taking CHEIs had significantly less positive expression of DLB-PCI2 compared to those who were not these agents (p = 0.003), despite the fact that the relationship between DLB-PCI2 expression and our primary clinical and objective measures of attention and FC (CAF, CRT, and CRT(SD)) was not confounded by CHEI use (Table 4).

Therefore, we hypothesized that DLB-PCI2 expression was acting as a mediator variable between CHEI use and our primary measures of FC and attentional function. Indeed, after controlling for DLB-PCI2 expression, the partial correlation between CHEI use and CAF, CRT, and CRT(SD) was reduced to non-significance (r = −0.14, p = 0.59; r = −0.13, p = 0.60; r = −0.16, p = 0.54, respectively), suggesting a strong mediation role for the expression of DLB-PCI2 in modulating CHEI use and improvements in CAF, CRT, and CRT(SD).

## Discussion

We found that in DLB patients, a single covariant perfusion network was positively associated with cognitive and attentional dysfunction as well as clinically and objectively measured FC and bradykinesia. This DLB-PCI2 network included covariant activity in both motor (tendency to increased activity) and associative parietal and parieto-temporal areas (tendency to decreased activity) bilaterally. No such networks were evident in those with AD patients, reinforcing the conclusion that FC and cognitive–attentional dysfunction in DLB have a specific underlying pathophysiological origin, which may relate, in part, to activity in both posterior cortical and motor system networks.

In contrast, PCI-1 in both DLB and AD, and PCI-2 in AD did not associate with our primary clinical variables. It may be that these perfusion patterns relate to other disease factors not measured in the present study. Certainly, the PCI-1 in both DLB and AD demonstrated perfusion changes which included periventricular regions and thus these PCIs may relate more to the gross, structural, atrophic changes rather than more nuanced, functional changes that associate with clinical symptoms or cognition. In addition, it is notable that the reported metabolic PCI which most strongly related cognition in PD (see below) was in fact the second and not the first PCI (Huang *et al*., 2007), similar to our findings here.

### 

#### DLB-PCI2: similarities and differences compared to other covariant lewy body disease networks

In PD, two covariant PET-related metabolic networks have been reported including a Parkinson's disease-related pattern (PDRP) and a Parkinson's disease-related cognitive pattern (PDCP, Eckert *et al*., 2007a; Huang *et al*., 2007). The former correlates with the severity and duration of motor symptoms in PD (Eidelberg *et al*., 1995; Lozza *et al*., 2004; Asanuma *et al*., 2006) and the latter is associated with neuropsychological performance in non-demented PD and PD with mild cognitive impairment (Lozza *et al*., 2004; Huang *et al*., 2007). The PDRP is characterized by relative increases in metabolic activity in pallidothalamic, pontocerebellar, and motor cortical and SMA metabolic activity with covariant decreases in lateral premotor and posterior parietal areas. In contrast, the PDCP shows increased metabolic activity in the cerebellum and dentate nuclei with relative decreases in the prefrontal and parietal cortex. Both the PDRP and the PDCP in PD share overlapping topology but are orthogonal in their associations with motor and cognitive deficits. In contrast, in the present data in DLB patients, there was a convergence of some aspects of motor deficit (bradykinesia) and cognitive deficits onto one perfusion network. Differences between the DLB-PCI2 and the PD-related metabolic networks in terms of the topology and direction of covariance may be explained by methodological differences between SPECT perfusion and PET metabolic imaging although given that univariate perfusion and metabolic patterns are broadly similar in DLB (Colloby *et al*., 2002, 2013; Abe *et al*., 2003), this is less likely to be factor. Alternatively, the differences between DLB-PCI2 and the PD metabolic networks could be the result of the more global and severe nature of the cognitive impairment in DLB compared to PD (e.g. Huang *et al*. study MMSE 28.3 ± 2.1 vs. MMSE 16.2 ± 5.6 in present study), as well as intrinsic differences in motor phenotype (e.g. Huang *et al*. (2007) study, UPDRS was 34.3 ± 18 vs. 26.6 ± 15.3 in present study) and levels of dopaminergic medication use between the two diseases.

On the other hand, the DLB-PCI2 network shared some topological characteristics seen in both PD-related metabolic patterns including relative SMA and cerebellar increases analogous to those seen in the PDRP as well as decreases in parietal areas which are evident in the PDCP. Therefore, a unifying explanation for the overlap in some areas, yet difference in others, between DLB-PCI2 and the PD network patterns may be that the topology of DLB-PCI2 represents the convergence of both motor and cognitive deficits onto one network after the development of more advanced cortical Lewy body pathology although in the present study while we did not see any association between disease duration and DLB-PCI2 expression. A proper analysis of covariant perfusion or metabolic networks longitudinally in cohorts of early stage DLB and PD patients who develop dementia would need to be carried out to clarify this hypothesis.

#### DLB-PCI2 and association with cognitive measures in dlb

DLB-PCI2 expression negatively correlated in DLB patients with global cognitive function in terms of the MMSE and CAMCOG. In addition, CAMCOG subscore scales of orientation, attention, and calculation negatively correlated with DLB-PCI2; these are all domains which are dependent upon attentional function, thus suggesting this is the main driver for the association between cognitive dysfunction and DLB-PCI2 expression. Of note, there was no clear association between DLB-PCI2 and the memory CAMCOG subscale. This is not unsurprising given that the topological distribution of DLB-PCI2 did not include medial temporal lobe structures which are more likely to be associated with deficits in this subscore. However, there was no strong association between DLB-PCI2 and the CAMCOG executive subscale score and while this might be explained by the relative lack of involvement of the frontal lobe in the DLB-PCI2, controlling for age improved the negative correlation between the CAMCOG executive subscore and DLB-PCI2 expression (although this was still only a trend association). This observation in conjunction with the fact that there was no association between DLB-PCI2 expression and age (r = −0.02, p = 0.95) may tentatively suggest that both age and DLB-PCI2 jointly affect executive function via similar mechanisms e.g. a deterioration in frontostriatal activity.

Nevertheless, revised versions of the CAMCOG executive (or memory) subscores have been critiqued in terms of their validity (Kessels *et al*., 2009) and a specific limitation of the present study was that detailed neuropsychological testing of executive function was not carried out which might have helped elaborate more clearly what elements of cognition and executive function (aside from attention) were related (or not) to DLB-PCI2.

#### DLB-PCI2 and association with cognitive fluctuations in dlb

The observation that a lesion in one cortical area does not necessarily lead to significant alterations in arousal or consciousness supports the conjecture that FC and arousal (Mesulam, 1981; Steriade, 2006) are unlikely to arise from discrete topographic areas but rather are likely to arise from a widely distributed and interconnected network. The positive association between FC clinically and the DLB-PCI2 corroborates this as the multivariate nature of the applied SCA is sensitive to dysfunctions across a distributed system rather than specific nodal deficits.

Several nodes of the DLB-PCI2 receive substantive cholinergic innervation including the striatum, parietal cortex, and cerebellum and thus a link between cholinergic function, activity within the DLB-PCI2 network, and FC could be hypothesized. In support of this, DLB patients on CHEI displayed better reaction times on attentional tasks and less FC than those not on these agents, which is consistent with previous reports in the literature (Wesnes *et al*., 2002), and mediation of this effect appeared to be in part via DLB-PCI2. However, we are cautious in our interpretation given the cross-sectional nature of the study and the fact that only five DLBs at the time of imaging were on CHEI. In addition, it is feasible that the association between CHEI and DLB-PCI2 as well its association with FC and attentional measures could be affected by a clinical treatment selection bias, although one might expect those initiated on CHEI to have more cognitive impairment which did not appear to be the case (Supplementary Table 1).

Other distributed cortical systems reliant on different neurotransmitters may be responsible for arousal and attention may be compromised and contribute to the development of FC; in particular, it has been posited that non-cholinergic systems including dopamine (particularly in PDD) and noradrenergic systems as well as structures that mediate arousal and circadian function such as the hypothalamus and midbrain may have a role (Francis *et al*., 2006). It is notable that the severity of bradykinesia, which is associated with hypodopaminergic states correlated with DLB-PCI2 expression. This with observation that DLB-PCI2 incorporated the striatum might suggest a dopaminergic role in certain aspects of attention and FC in DLB, although we did not see any clear association between UPDRS total scores and DLB-PCI2 expression. Rather our data tend to favor the established argument that cholinergic function has a more predominant role with regard to attentional function in DLB (Wesnes *et al*., 2002).

### DLB-PCI2 and association with objective attention measures

Reaction time delays in CRT as well as greater fluctuations in CRT have been reported in DLB and PDD (Ballard *et al*., 2002) and we confirmed this association and also found significant associations between DLB-PCI2 expression and CRT and CRT(SD).

Visuo-perceptual motor tasks have been suggested to decompose into four discrete temporally ordered elements (Donders, 1869): stimulus detection, stimulus discrimination, response selection, and motor execution and it is likely that CRT reaction time is dependent upon all these factors. The association between CRT and its variability with DLB-PCI2 as well as the topographic alignment of the DLB-PCI2 onto areas affiliated to perceptual processing (parietal) and motor processing (SMA, cerebellum, and striatum) may suggest that these specific processing components (and their dysfunction) involved in the CRT may be relatively more important in the etiology of attentional dysfunction and FC in DLB.

However given we only examined CRT and CRT(SD) in the present study, we must qualify this statement with the caveat that it is unclear whether DLB-PCI2 specifically relates to dysfunction in this aspect of attention or more broadly to other attentional components (e.g. vigilance), although it is notable that CRT and CRT(SD), which include all components of visuo-perceptual motor processing and their variability, are the strongest objective attentional measures which associate with FC. Therefore, it is likely that the topographic regions involved in DLB-PCI2 perfusion network are areas which may have a role in FC in DLB.

### Understanding the topography of DLB-PCI2

Evidence for structural connectivity between pre-SMA/SMA, basal ganglia, and cerebellum provides a basis to support the observed perfusion covariance seen between these areas in the DLB-PCI2. The widespread relative reduction in parietal perfusion is consistent with observed posterior, parietal EEG (Bonanni *et al*., 2008), and resting state network abnormalities in DLB (Franciotti *et al*., 2013), which are known to associate with the severity of FC and the putative role these regions have in attentional function. The relative increases in the motor system are perhaps more difficult to explain but might reflect a compensatory activity in response to a relative cholinergic/dopaminergic deficit. In addition, while these areas subserve motor functions (e.g. the fronto-striatal SMA–putamen motor loop), they also have significant roles in cognition (Middleton and Strick, 2000). For example, the pre-SMA, which receives inputs from the cerebellum and basal ganglia (Akkal *et al*., 2007), has been associated with cognition and attention (e.g. Macar *et al*., 2006), and functional disconnection between the prefrontal cortex and the SMA in PD is associated with impaired attention to action performance (Rowe *et al*., 2002). The SMA may also be particularly pertinent to reaction time tasks given its putative activation with non-sequential and stimulus-cued movement (Picard and Strick, 2003) and its activation in fMRI -related CRT tasks (Winterer *et al*., 2002).

PD patients have abnormalities in time estimation (Pastor *et al*., 1992) and dopamine-dependent basal ganglia dysfunction leads to abnormal temporal processing in the millisecond to seconds range (so called interval timing) (Jones *et al*., 2008) and it is notable that perceptual timing models include elements of a motor network which overlaps with DLB-PCI2 such as the cerebellum, basal ganglia, pre-SMA, and SMA (Macar *et al*., 2006; Teki *et al*., 2011). Therefore, an intriguing question is whether slowed task reaction time and its variability, as well as FC in DLB, intrinsically relate to aberrant processing of time via motor networks. Further work with task-related functional neuroimaging would be required to test the veracity of this hypothesis.

The DLB-PCI2 also included increased midbrain (possibly inclusive of superior colliculus) and medial cerebellar activity with decreased parietal lobe activity which may hint at dysfunctions in networks associated with attention shifting/spatial orienting (Posner *et al*., 1982; Rafal *et al*., 1988) although given the limits of spatial resolution of SPECT, this can only be a speculative comment.

Surprisingly, we did not see any alteration in activity within thalamic areas despite prior clinicopathological and neuroimaging evidences implicating this area in FC in DLB (O'Brien *et al*., 2005; Pimlott *et al*., 2006; Piggott *et al*., 2007). A SCA approach only considers areas which covary in activity and thus it is feasible that any absolute perfusion changes related to FC within this structure do not associate with perfusion alterations elsewhere although it is notable when we carried out a *post hoc* univariate region of interest analysis of thalamic SPECT perfusion and its relationship to FC, we failed to find any significant correlations in the present cohort (data not shown).

We also did not see any significant topographic overlap between DLB-PCI2 and the default mode network (DMN) which associates with attentional task performance (Weissman *et al*., 2006). Part of this may be due to the fact that our spatial resolution is lower with SPECT than PET for example, and thus perhaps activity which appeared localized to the pre-SMA/SMA may actually have intruded into the anterior cingulate cortex (ACC), an established hub in the DMN. However, recent data suggest that the DMN function is actually preserved in DLB patients (Franciotti *et al*., 2013) (even those with severe FC), thus suggesting that the DMN may not be a major player in terms of FC in DLB, although perhaps other resting state networks, e.g. ventral or dorsal attention network, might be more salient.

Strengths of the study include the careful diagnostic work-up of patients, as well as neuropathologic confirmation in a minority. A major limitation of the present study is the lower resolution of SPECT perfusion imaging in comparison to PET metabolism imaging. While our covariant networks maintained statistical robustness, this issue still restricts the spatial accuracy of delimiting topographic structures involved in perfusion networks. Finally, it is important to note that covariant analyses provide a relativistic measure between different cortical regions rather than absolute quantification; thus, it cannot determine which areas or regions in the network are specifically “normal” versus “abnormal” in function and how these relate to the clinical phenotype or symptoms, only that the overall network pattern itself is intrinsically associated with these disease features. Nevertheless, it is clear that multivariate approaches which are sympathetic to the network dynamics of the brain offer an additional means by which to examine pathophysiological processes in DLB.

## Conclusions

Using SCA applied to SPECT perfusion imaging, we provide evidence of a DLB-specific covariant perfusion network (which presumptively is an expression of an underlying neural network) that encompasses bilateral motor areas and the parietal and parieto-temporal cortices and which are strongly associated with cognition and FC.

Attentional performance as measured by the CRT in DLB and FC appears to map onto a combined motor and cognitive network. While this network does not explain the cause of FC or attentional dysfunction in its entirety in DLB, our findings suggest a link between FC and cognition via a combined cognitive-motor network, which in part might be influenced by distributed cholinergic dysfunction.

## Conflict of interest

None.

## Description of authors' roles

J.-P. Taylor was responsible for the inception of the research question, study design, data analysis, and prepared the paper. S. J. Colloby developed the multivariate analysis approaches and helped review and revise drafts of the paper. I. G. McKeith was involved in the patient recruitment, participant diagnostic rating, and made comments on the paper. J. T. O'Brien was involved in the participant recruitment, participant diagnostic rating, and helped review and revise drafts of the paper. In addition, he acted as a fellowship sponsor to J.-P. Taylor.
